# Nectin-4 reduces T cell effector function and is a therapeutic target in pancreatic cancer

**DOI:** 10.1172/jci.insight.194290

**Published:** 2025-12-09

**Authors:** Max Heiduk, Carolin Beer, Sarah Cronjaeger, Emily A. Kawaler, Ulrich Sommer, Franziska Baenke, David Digomann, Loreen Natusch Bufe, Charlotte Reiche, Jessica Glück, Franziska Hoffmann, Sungsik Kim, Daniel E. Stange, Diane M. Simeone, Jürgen Weitz, Lena Seifert, Adrian M. Seifert

**Affiliations:** 1 Department of Visceral, Thoracic and Vascular Surgery, University Hospital Carl Gustav Carus, Technische Universität Dresden, Dresden, Germany.; 2 National Center for Tumor Diseases (NCT), Dresden, Germany.; 3 German Cancer Research Center (DKFZ), Heidelberg, Germany.; 4 Faculty of Medicine and University Hospital Carl Gustav Carus, Technische Universität Dresden, Dresden, Germany.; 5 Helmholtz-Zentrum Dresden - Rossendorf (HZDR), Dresden, Germany.; 6 Perlmutter Cancer Center, NYU Langone Health, New York, New York, USA.; 7 Institute of Pathology, Faculty of Medicine Carl Gustav Carus, and; 8 National Center for Tumor Diseases (NCT), Biobank Dresden, University Hospital Carl Gustav Carus, Technische Universität Dresden, Dresden, Germany.; 9 German Cancer Consortium (DKTK), Partner Site Dresden, German Cancer Research Center (DKFZ), Heidelberg, Germany.; 10 Department of Surgery, Moores Cancer Center, UC San Diego Health, San Diego, California, USA.; 11 Else Kröner Clinician Scientist Professor for Translational Tumor Immunological Research, Dresden, Germany.

**Keywords:** Gastroenterology, Oncology, Cancer, T cells

## Abstract

Pancreatic ductal adenocarcinoma (PDAC) has a dismal prognosis, and current therapies show limited efficacy. Ligands and receptors of the TIGIT axis were analyzed using multicolor flow cytometry of tumor and blood samples, IHC from primary tumors, and single-cell RNA-Seq from primary tumors and liver metastasis from patients with various stages of PDAC. The effect of soluble and plate-bound Nectin-4 on T cell function was tested in vitro. Furthermore, patient-derived PDAC organoids were treated with the standard-of-care therapies FOLFIRINOX, gemcitabine plus paclitaxel, or the antibody-drug conjugate enfortumab vedotin. TIGIT expression was increased on tumor-infiltrating conventional T cells and Tregs compared with T cells from matched blood. Nectin-4 but not CD155 expression was associated with poor outcome. Nectin-4 was exclusively expressed by tumor cells and correlated with low immune infiltration. Notably, Nectin-4 inhibited T cell effector cytokine production in vitro. Targeting Nectin-4 with the antibody-drug conjugate enfortumab vedotin inhibited tumor growth in multiple patient-derived PDAC organoids. Collectively, our data underscore Nectin-4 as a potential novel therapeutic target and provide the rationale to test this agent in patients with PDAC.

## Abstract 2

Nectin-4 correlates with low immune infiltration, poor outcome, and reduces T cell effector function. Nectin-4 is a therapeutic target in pancreatic cancer and can be effectively targeted with antibody-drug conjugates.

## Graphical abstract

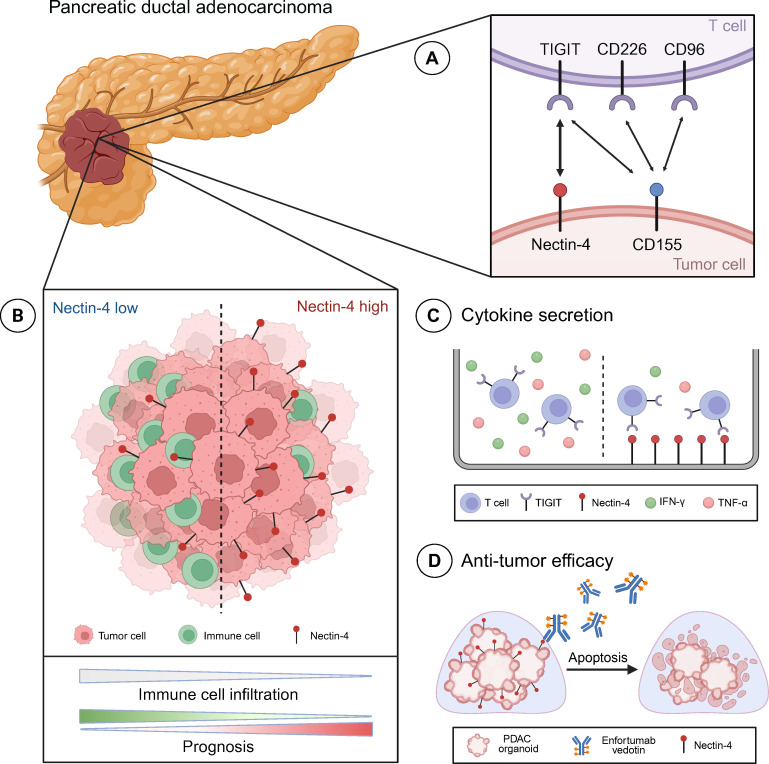

## Introduction

Pancreatic ductal adenocarcinoma (PDAC) is one of the deadliest malignancies, with a 5-year survival rate across all tumor stages of only 13% (1). Immunotherapies targeting immune checkpoint receptors (ICRs) have revolutionized cancer treatment but have not yet shown efficacy in PDAC due to a highly immunosuppressive tumor immune microenvironment and poor immunogenicity (2). Intratumoral T cells in PDAC are mostly dysfunctional and exhausted (3). Nevertheless, increased infiltration of effector T cells and a proinflammatory, antitumorigenic immune infiltrate are associated with improved survival in PDAC, suggesting potential for immunotherapies (4). The TIGIT axis comprises a network of lymphocyte-expressed ICRs, such as TIGIT, CD226, and CD96, which interact with the ligands CD155 and members of the Nectin family (5). Recently, we identified TIGIT as a marker of exhausted CD8^+^, conventional CD4^+^ T cells (Tconvs), and immunosuppressive Tregs in PDAC (6–8). In a neoantigen-expressing PDAC mouse model, CD155 overexpression led to immune evasion, which was overcome with combinational immunotherapy of anti-PD-1, anti-TIGIT, and agonistic CD40 antibody treatment (9). While previous studies have mainly focused on TIGIT and CD155 as one of its ligands, a comprehensive understanding of the axis is still needed to evaluate its potential for future immunotherapeutic strategies in PDAC. Our data reshape the understanding of the TIGIT axis in PDAC by underscoring Nectin-4 expression as a major mechanism of immune escape and therapeutic target in PDAC.

## Results

### TIGIT, CD226, and CD96 are expressed by tumor-infiltrating T cells.

**Figure 1:**
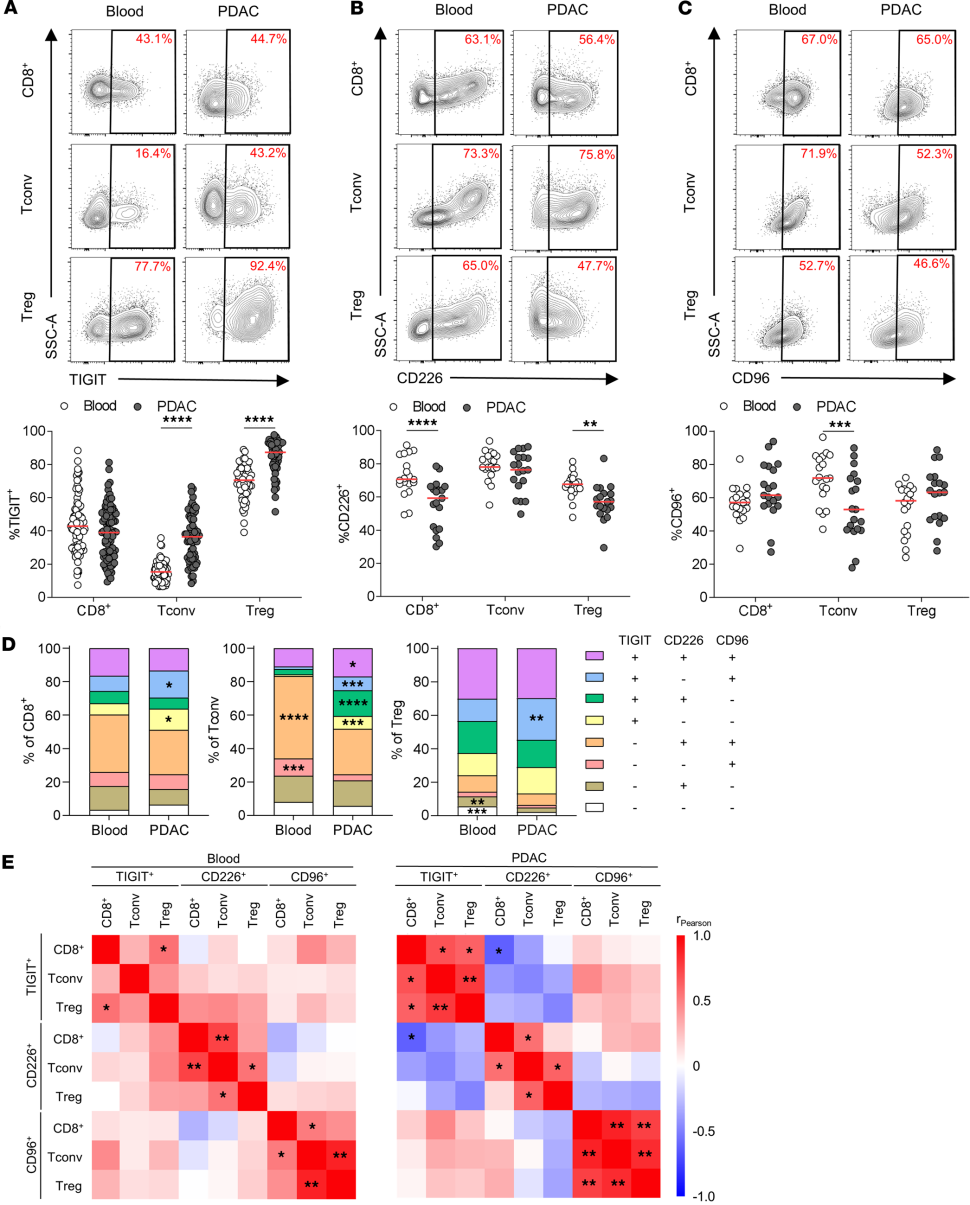
TIGIT, CD226, and CD96 are expressed by tumor-infiltrating T cells. (**A**) Representative contour flow plots for expression of TIGIT and percentage of TIGIT by CD8^+^ T cells, Tconv, and Treg in matched blood and PDAC (*n* = 84). (**B**) Representative contour flow plots for expression of CD226 and percentage of CD226 by CD8^+^ T cells, Tconv and Treg in matched blood and PDAC (*n* = 19). (**C**) Representative contour flow plots for expression of CD96 and percentage of CD96 by CD8^+^ T cells, Tconv, and Treg in matched blood and PDAC (*n* = 19). Each point represents data from 1 patient. Medians are shown as horizontal red lines. Paired 2-tailed *t* tests with Holm-Šídák correction were used. (**D**) Stacked columns showing the mean coexpression of TIGIT, CD226, and CD96 among CD8^+^ T cells, Tconv, and Treg (from left to right) in blood versus PDAC. *P < 0.05; **P < 0.01; ***P < 0.001; ****P < 0.0001. (**E**) Heatmap showing Pearson correlation coefficient (r_Pearson_) for correlation between ICR expressions by indicated T cell subsets in blood (left) and PDAC (right). To correct for multiple comparisons in correlation analysis, significance levels were set at *P < 0.01; **P < 0.001.

To identify the distribution of receptors and ligands of the TIGIT axis in the pancreatic tumor microenvironment, we analyzed the known receptors of the TIGIT axis. TIGIT, CD226, and CD96 are important immune regulatory receptors involved in modulating T cell activity in antitumor immunity (10). The expression of these receptors by T cells isolated from blood and matched PDAC tumor samples was analyzed by multicolor flow cytometry (Figure 1, A–C). Patient characteristics are shown in Supplemental Table 1 (supplemental material available online with this article; https://doi.org/10.1172/jci.insight.194290DS1). TIGIT was highly expressed by Treg in blood and PDAC and significantly increased in Tconv and Treg in PDAC compared with blood (Figure 1A). CD226 expression by intratumoral CD8^+^ T cells and Treg (Figure 1B) and CD96 expression by Tconv was significantly reduced in PDAC (Figure 1C). While the distribution of the ICR coexpressing subsets of CD8^+^ T cells was largely similar between blood and PDAC, TIGIT^–^CD226^+^CD96^+^ cells constituted the main subset among blood Tconv but were significantly decreased in PDAC (Figure 1D). Cells were significantly increased among PDAC Tconv. TIGIT^+^CD226^–^CD96^+^ Treg were increased in PDAC, whereas Treg with no expression of these ICRs were almost absent in PDAC. Notably, TIGIT and CD96 expression showed a strong positive correlation between T cell subsets in blood and PDAC (Figure 1E and Supplemental Tables 2 and 3). CD226 and TIGIT expression by CD8^+^ T cells in PDAC correlated negatively. Furthermore, different transcription factors were investigated to assess their association with ICR expression (Supplemental Figure 1, D–F). Representative gating is shown in Supplemental Figure 1, A–C. The proliferation marker Ki-67 was particularly high among CD226^+^ Treg but also significantly increased among TIGIT^+^CD8^+^ T cells and TIGIT^+^ Tconv, indicating an increased proliferation of these subsets (Supplemental Figure 1D). Eomesodermin (Eomes), which drives CD8^+^ T cell exhaustion (11), was significantly higher in TIGIT^+^ but was lower in CD226^+^ and CD96^+^ CD8^+^ T cells (Supplemental Figure 1E). Most interestingly, GATA3, a transcription factor known to drive the differentiation toward a rather antiinflammatory and tumor-protective phenotype (12), was significantly increased among TIGIT^+^CD8^+^ T cells and Tconv but was not associated with expression of the other ICRs (Supplemental Figure 1F). In the blood, T cells present significantly different phenotypes based on their ICR expression, with Ki-67, Eomes, and GATA3 expression being associated with TIGIT positivity.

### Nectin-4 expression is associated with poor outcome in PDAC.

**Figure 2:**
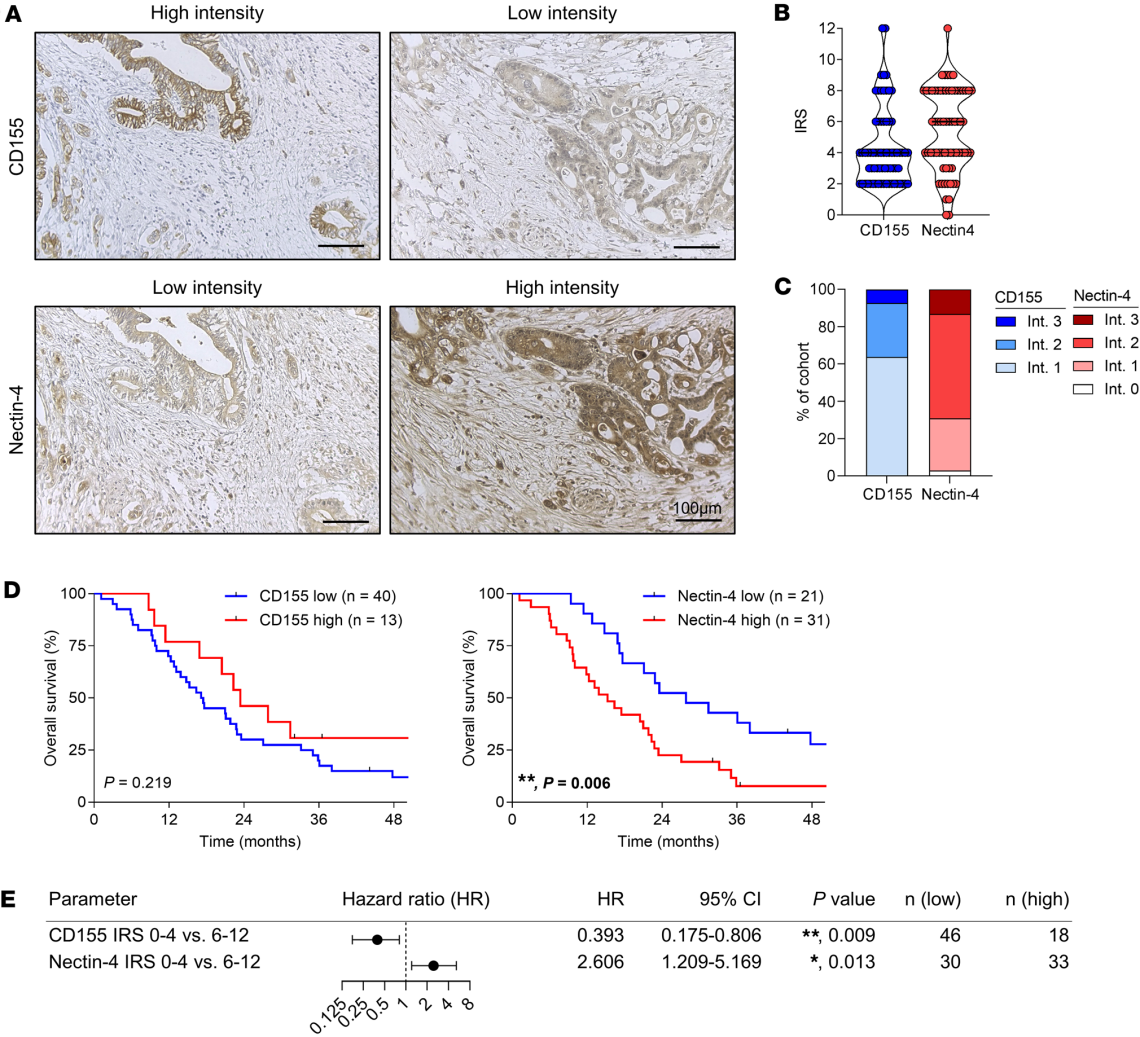
Nectin-4 expression is associated with poor outcome in PDAC. (**A**) Representative images of CD155 (top) and Nectin-4 (bottom) IHC staining with low and high intensity. Scale bar: 100 μm. (**B**) Dot plot showing the distribution of immune reactive scores (IRS; CD155, *n* = 69; Nectin-4, *n* = 68). (**C**) Stacked columns depicting the proportion of patients with CD155 and Nectin-4 expression according to intensity. (**D**) Kaplan-Meier analysis of overall survival of R0-resected patients with PDAC according to low or high CD155 (left) or Nectin-4 (right) expression. *P* values of log rank test are indicated. (**E**) Table and forest plot depicting survival hazard ratios (HR) with 95% CI of CD155 and Nectin-4 IRS in multivariate Cox proportional hazards regression analysis including both R0- and R1-resected patients, shown as a function of clinicopathological parameters. *P < 0.05; **P < 0.01.

Subsequently, the expression and prognostic relevance of the TIGIT ligands, namely *PVR* (CD155), *PVRL1* (Nectin-1), *PVRL2* (Nectin-2), *PVRL3* (Nectin-3), and *PVRL4* (Nectin-4) was examined using the TCGA dataset (Supplemental Figure 2, A and B). Intriguingly, high *PVR* expression was associated with significantly improved overall survival in PDAC, whereas *PVR* and *PVRL1* expression was linked to reduced survival in hepatocellular carcinoma. Most interestingly, while *PVRL4* expression was associated with favorable survival in gastric cancer, it was associated with reduced survival in PDAC. These differences suggest disease-specific prognostic relevance of these proteins. Based on these data, our analyses focused on CD155 and Nectin-4. The correlation between *PVRL4* expression and that of several genes of interest was investigated within the TCGA data set (Supplemental Figure 2C). *PVRL4* showed a positive correlation with *PVRL2* but was negatively associated with immune cell–related genes, particularly T and NK cell genes, and to a lesser extent with different myeloid cell genes. The expression of *PVRL4* and ICR genes, which are predominantly expressed by T cells, consistently showed a negative correlation. The lowest correlation coefficient between *PVRL4* and any immune-associated gene was with *CD226* (*r* = 0.560). In contrast, *PVRL4* correlated positively with *LGALS3* (Galectin-3), another ligand known to be involved in immune escape in PDAC (13), and 2 epithelial genes representative of ductal tumor cells. There was no or a negative association with genes related to fibroblasts or extracellular matrix formation, indicating that Nectin-4 is mainly expressed by tumor cells rather than within the stromal compartment. *PVRL2* also positively correlated with *PVR* and was the only ligand that showed a minor negative correlation with immune-related genes (Supplemental Table 4). By IHC, tumor cells showed a distinct CD155 and Nectin-4 staining pattern (Figure 2A). No significant differences in expression between the tumor core and periphery were detected, nor was any association observed with other histological features. The intensity and percentage of positive tumor cells were scored to calculate the immune reactive scores (IRS) (Figure 2B). An IRS of 0–4 was classified to be low and an IRS of 6–12 as high expression. Of note, Nectin-4 staining was absent in only 2 of the 68 patients (2.9%). Approximately 70% of PDAC samples had high and very high Nectin-4 expression, whereas CD155 had a low intensity in more than 60% of the samples (Figure 2C). CD155 expression was not significantly associated with prognosis in the univariate analysis, but high Nectin-4 expression was strongly associated with reduced survival of patients with PDAC (Figure 2D). Notably, in the multivariate analysis, high CD155 expression was significantly associated with increased survival, while high Nectin-4 expression was a significant risk for reduced survival (Figure 2E). No significant association was observed between expression of either protein and any clinicopathologic characteristics (Supplemental Table 5).

### Nectin-4 expression is associated with reduced immune cell infiltration in PDAC.

**Figure 3:**
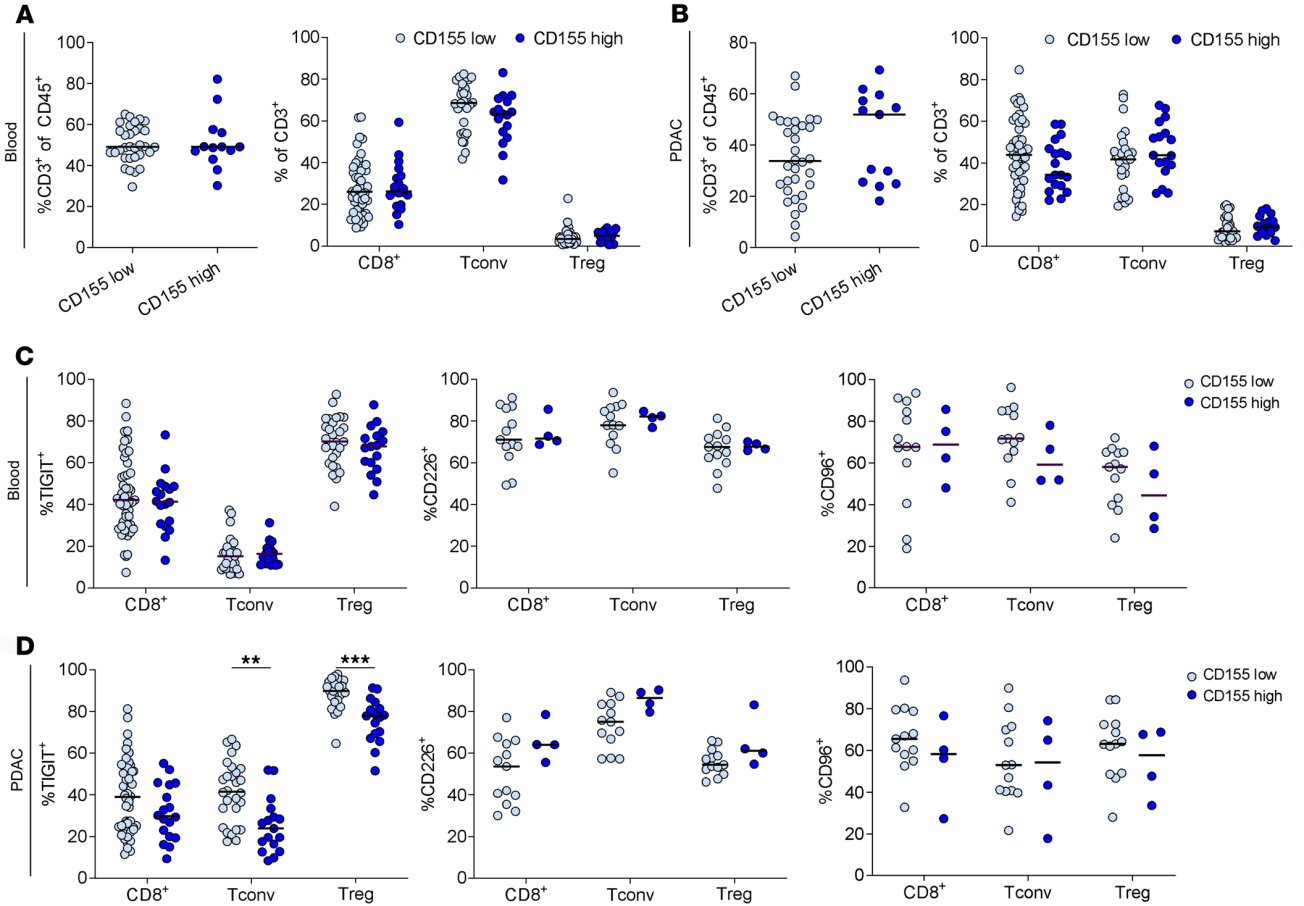
CD155 expression is associated with reduced TIGIT expression by PDAC-infiltrating Tconv and Tregs. (**A** and **B**) Percentage of T cells among all immune cells (left) and percentage of indicated T cell subsets among all T cells (right) in blood (**A**) and PDAC (**B**) for low and high CD155 expression (*n* = 69). (**C** and **D**) Percentage of TIGIT, CD226 and CD96 expression (from left to right) for indicated T cell subsets in blood (**C**) and PDAC (**D**) for low and high CD155 expression. Each point represents data from 1 patient. Medians are shown as horizontal lines. Unpaired 2-tailed *t* tests with Holm-Šídák correction. **P < 0.01; ***P < 0.001.

**Figure 4:**
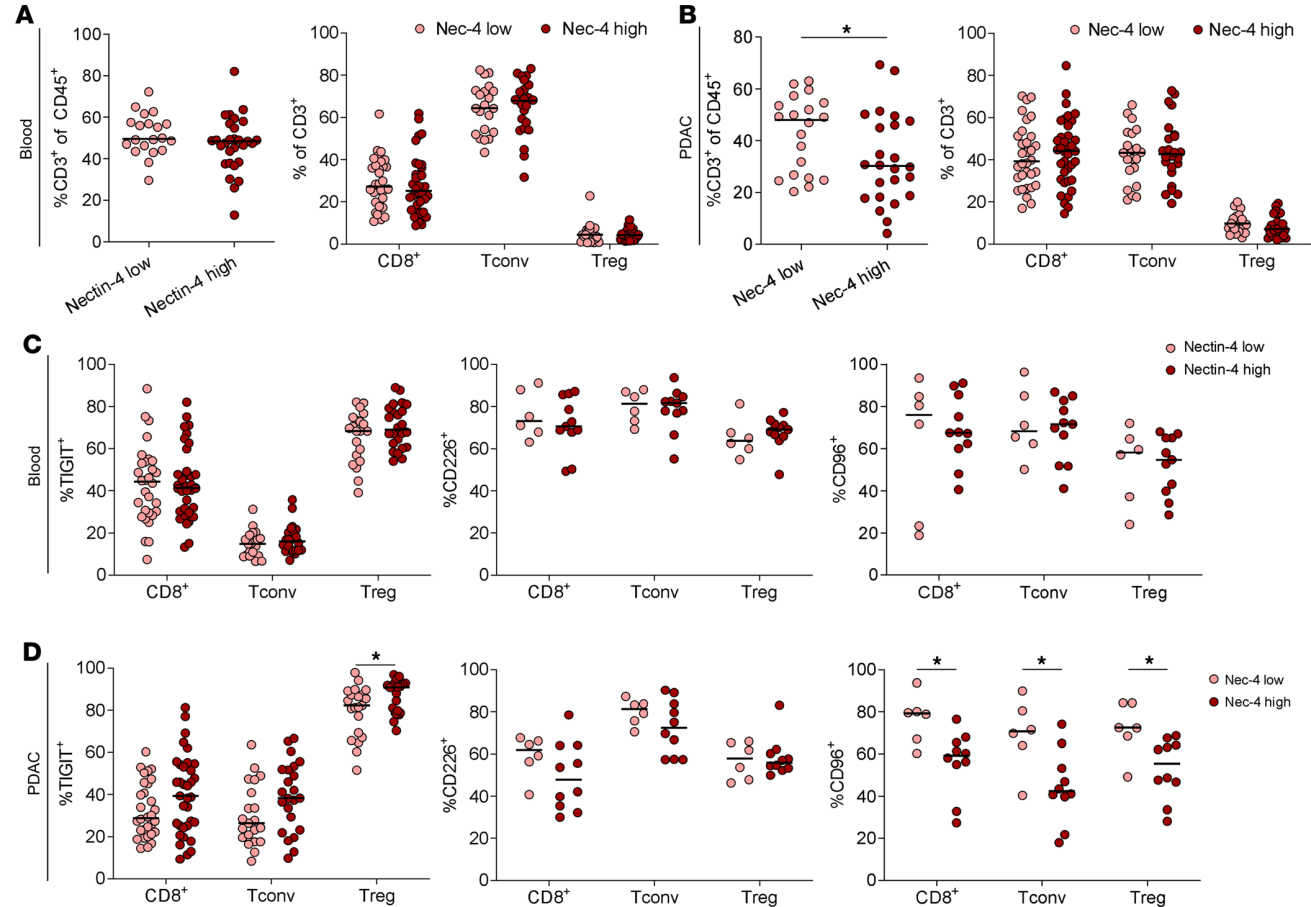
Nectin-4 expression is associated with reduced immune cell infiltration in PDAC. (**A** and **B**) Percentage of T cells among all immune cells (left) and percentage of indicated T cell subsets among all T cells (right) in blood (**A**) and PDAC (**B**) for low and high Nectin-4 expression (*n* = 68). (**C** and **D**) Percentage of TIGIT, CD226 and CD96 expression (from left to right) for indicated T cell subsets in blood (**C**) and PDAC (**D**) for low and high Nectin-4 expression. Each point represents data from 1 patient. Medians are shown as horizontal lines. Unpaired 2-tailed *t* tests with Holm-Šídák correction. *P < 0.05.

Next, the correlation between ligand expression and intratumoral T cell infiltration and phenotypes was evaluated. CD155 expression was not associated with the frequency of T cell subsets in blood (Figure 3A) or tumor (Figure 3B). Furthermore, the expression of TIGIT, CD226, and CD96 in blood T cell subsets (Figure 3C) was independent of CD155 tumor tissue expression, but PDAC-infiltrating Tconv and Treg showed decreased TIGIT expression in CD155^hi^ PDAC (Figure 3D). While the PDAC cohort with high CD155 expression was small, no significant difference in CD226 and CD96 expression was detected compared with CD155^lo^ PDAC samples. All intratumoral T cell subsets in the CD155^hi^ cohort showed a trend toward increased CD226 expression. Strikingly, Nectin-4^hi^ tumors displayed no differences in the general T cell frequency and subset frequency in the blood (Figure 4A), but a significantly reduced frequency of T cells among all immune cells in the tumor, but without alterations in T cell subset composition (Figure 4B). Again, high Nectin-4 expression was not associated with changes of the ICR expression by blood T cell subsets (Figure 4C), but PDAC-infiltrating Treg showed increased TIGIT expression, with a similar trend for CD8^+^ T cells and Tconv (Figure 4D). Intratumoral T cells from Nectin-4^hi^ PDAC had slightly reduced CD226 expression but significantly reduced CD96 expression among all T cell subsets.

### Nectin-4 is exclusively expressed by tumor cells in PDAC.

**Figure 5:**
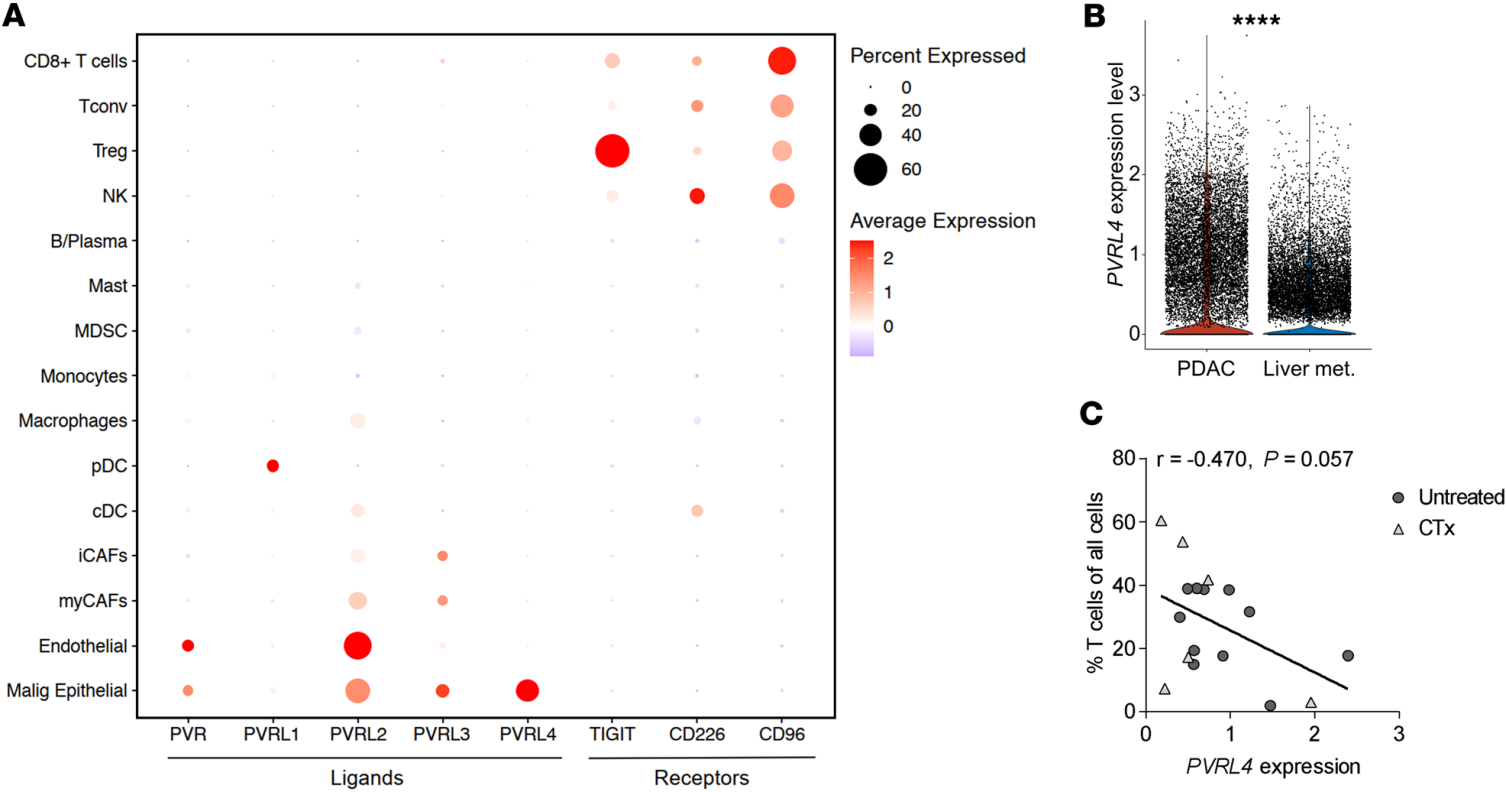
Nectin-4 is exclusively expressed by tumor cells in PDAC. (**A**) Dot plot depicting gene expression of TIGIT family receptors and ligands in several compartments within human primary PDAC (*n* = 17). The dot size represents the percentage of cells expressing the gene, and the color represents the average expression within those cells. (**B**) Violin plot of *PVRL4* expression in all malignant epithelial cells in primary PDAC (*n* = 11) compared with PDAC liver metastases (*n* = 9) from treatment-naive patients. (**C**) Scatter plot showing the correlation between *PVRL4* expression in malignant epithelial cells and percentage of T cells among all analyzed cells per sample in treatment-naive (*n* = 11) and chemotherapeutically-treated (*n* = 6) primary PDAC. Pearson correlation coefficients and *P* values are depicted. Each dot represents 1 sample. Wilcoxon signed-rank test for comparison of expression levels. ****P < 0.0001.

To further validate the relevance of Nectin-4 in PDAC, we analyzed 17 primary tumor and 9 liver metastasis samples using scRNA-Seq (Figure 5 and Supplemental Figure 3, A–C). *TIGIT*, *CD226,* and *CD96* were expressed by all T cell subsets and NK cells to varying degrees, with CD8^+^ T cells exhibiting the highest expression of *CD96*, while Treg had high *TIGIT* expression in PDAC (Figure 5A). Notably, *PVRL4* (Nectin-4) was expressed exclusively by tumor cells, while *PVRL2* (Nectin-2) was expressed by various cell types, including CAFs, myeloid, malignant epithelial, and endothelial cells. A low percentage of endothelial and tumor cells expressed *PVR* (CD155). Expression patterns were broadly similar between treatment-naive PDAC, PDAC treated with chemotherapy, and treatment-naive PDAC liver metastases (Supplemental Figure 3A). Interestingly, while *PVR* and *PVRL4* were also expressed by tumor cells in PDAC liver metastases, the expression of *PVR* was significantly increased in the metastatic cells (Supplemental Figure 3B), and *PVRL4* showed higher expression within the tumor cells from the primary tumor (Figure 5B). *PVR* and *PVRL4* expression correlated negatively in PDAC (Supplemental Figure 3C). Furthermore, a trend for a negative correlation between *PVRL4,* but not *PVR,* and the proportion of cells per sample in the T cell compartment, a proxy for lymphocyte infiltration, was observed (Figure 5C and Supplemental Figure 3C).

### Enfortumab vedotin has antitumor efficacy in PDAC PDOs.

**Figure 6:**
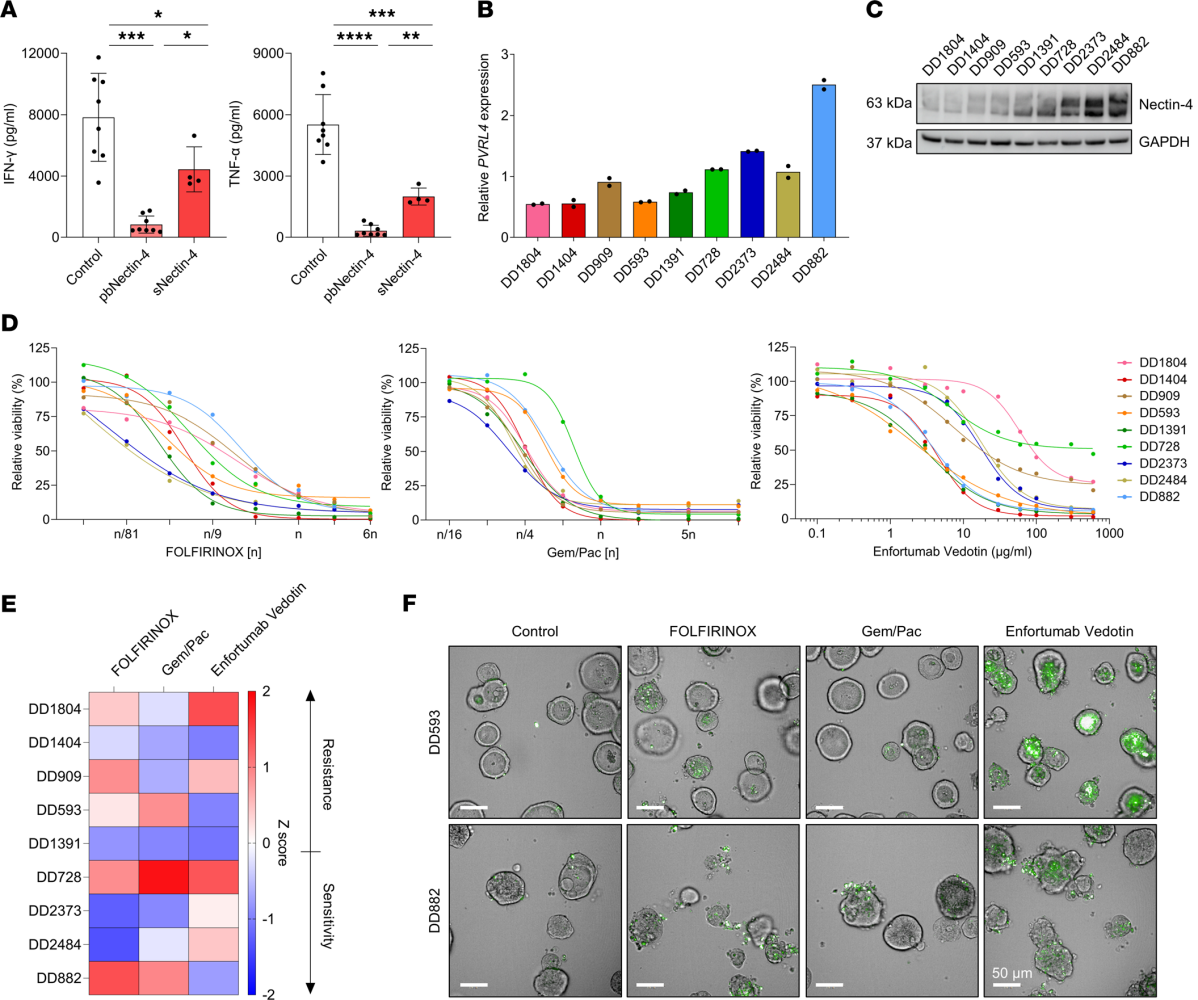
Enfortumab vedotin has antitumor efficacy in PDAC PDOs. (**A**) IFN-γ and TNF-α production by peripheral T cells from patients with PDAC after in vitro stimulation with anti-CD3 and anti-CD28 in the presence of plate-bound (pb, *n* = 8) or soluble (s, *n* = 4) Nectin-4. Each point represents data from 1 patient. Data are shown as mean ± SD. Unpaired 2-tailed *t* tests with Welch’s correction. *P < 0.05; **P < 0.001; ***P < 0.001; ****P < 0.0001. (**B**) Representative expression level of *PVRL4* by qPCR (bars indicate mean of technical duplicates). (**C**) Nectin-4 expression in PDAC PDOs by Western blot. (**D**) Dose response curves from PDAC PDOs treated with FOLFIRINOX, gemcitabine plus paclitaxel (Gem/Pac), or enfortumab vedotin. The relative viability in percentage at a given drug concentration of 2 independent biological replicates is shown. (**E**) *Z* scores generated from relative AUC from dose response curves from PDAC PDOs either treated with FOLFIRINOX, Gem/Pac, or enfortumab vedotin. (**F**) Representative images of 2 PDAC PDOs either treated with the standard regimen FOLFIRINOX, Gem/Pac, or enfortumab vedotin. PDOs were stained with caspase-3 dye profiling apoptosis (green) and imaged after 3 days. Scale bar: 50 μm.

Upon activation, T cells produce proinflammatory cytokines like IFN-γ and TNF-α, which are crucial for mediating antitumoral immune responses. To assess the effect of Nectin-4 on T cell function, we cultured activated T cells from patients with PDAC in the presence of plate-bound (pb) (*n* = 8) or soluble Nectin-4 (sNectin-4, *n* = 4). After 3 days, T cells cultured with pbNectin-4 exhibited a significant reduction of IFN-γ and TNF-α secretion, compared with the control without Nectin-4 (Figure 6A). In the presence of sNectin-4, cytokine expression was also significantly reduced but to a lesser extent. Next, we tested several patient-derived organoids (PDO) from patients with PDAC for their Nectin-4 expression using RT-qPCR (Figure 6B) and Western blotting (Figure 6C). PDAC PDOs showed varying *PVRL4* mRNA expression, which corresponded to Nectin-4 protein expression. Due to its tumor-specific and generally high expression in PDAC, Nectin-4 may be a potential therapeutic target. Therefore, we investigated the antitumor effiacacy of enfortumab vedotin (EV) in in vitro drug screens on PDAC PDOs. Therapeutic responses were compared with the effiacacy of the chemotherapy regimens FOLFIRINOX and gemcitabine plus paclitaxel (Gem/Pac). Here, a wide range of responses was observed for each treatment (Figure 6D). *Z* scores of relative AUCs were calculated to detect different response patterns within the same line, demonstrating drug-sensitivity to EV in 4 (DD593, DD882, DD1391, and DD1404) PDAC PDOs (Figure 6E). DD1391 and DD1404 showed sensitivity to all treatments, whereas DD728 was resistant. Interestingly, DD593 and DD882 exhibited resistance to either 1 or both chemotherapeutic regimens, while displaying sensitivity to EV. To further explore the effect of EV on the chemotherapy-resistant PDOs DD593 and DD882, we evaluated apoptosis by caspase-3 staining. Strikingly, after 3 days of incubation, DD593 and DD882 underwent early apoptosis upon treatment with EV, while treatment with FOLFIRINOX and Gem/Pac resulted in only a few apoptotic cells (Figure 6F). Collectively, EV demonstrated antitumoral efficacy in approximately 50% of PDAC PDOs.

## Discussion

TIGIT expression plays a central role in T cell exhaustion in human and murine PDAC (6, 9). Furthermore, Treg have a higher abundance of TIGIT in the blood and within the tumor, which is consistent with an attenuated signal transduction response in Treg in comparison to effector T cell subsets (7). TIGIT acts as a counterregulatory protein to CD226 by competing for binding to its primary ligand CD155 with higher affinity and by disrupting its homodimerization (5). Costimulation by CD226 is important for T cell antitumor function and is associated with a better response to immune checkpoint blockade in lung cancer and melanoma (14, 15), and it can even compensate for CD28 deficiency (16). Therefore, significantly reduced CD226 expression by intratumoral CD8^+^ T cells observed in PDAC suggests an impaired ability for crucial costimulation. Interestingly, TIGIT and CD226 expression showed a negative correlation, further highlighting their functional interplay. Studies that originally suggested the involvement of the TIGIT axis in PDAC immune evasion highlighted CD155 by predicting its interaction with TIGIT within scRNA-Seq data (6, 17). TIGIT/CD155 interaction promoted immune evasion in murine PDAC that was overcome by combinational immunotherapy, including TIGIT-blockade (9). Since murine TIGIT does not interact with murine Nectin-4 or other ligands, but only with murine CD155, classical mouse models are insufficient to study the TIGIT axis, and conclusions that can be drawn are limited (18, 19). Therefore, we solely analyzed human samples and assessed ligands and ICR beyond TIGIT and CD155 to identify critical components within the network. High Nectin-4 expression has been detected in various solid tumors and was associated with unfavorable prognosis in esophageal and gastric cancer (20–22). Nectin-4 has been proposed as a diagnostic biomarker in lung and metastatic breast cancer (23, 24). Strikingly, Nectin-4 expression correlated negatively with T cell infiltration in the TCGA, scRNA-Seq, and IHC/flow cytometry data sets. Nectin-4 was associated with increased TIGIT expression by PDAC-infiltrating T cells, but not in blood T cells, indicating potential Nectin-4–derived immunosuppression via the TIGIT axis, particularly in the PDAC tumor microenvironment. In contrast, tumor CD155 expression was associated with reduced TIGIT expression, suggesting an increased antitumor immunity in CD155 high tumors. CD155 may provide costimulation via interaction with CD226, whereas Nectin-4 appears to solely interact with TIGIT (5, 18). Our study provides important human data to expand our knowledge of the TIGIT axis in PDAC by suggesting that Nectin-4, rather than CD155, is the central ligand for TIGIT axis–mediated PDAC immune evasion by elucidating its potential as therapeutic target. Despite both serving as TIGIT ligands, their opposing prognostic effects suggest distinct immunomodulatory functions that warrant ligand-specific therapeutic targeting. When cultivated with Nectin-4, T cells produced significantly fewer effector cytokines. Interestingly, this effect was more pronounced with pb than soluble Nectin-4. TIGIT expression by T cells was similar across all patients, indicating that observed immunosuppressive effects were not caused by differential TIGIT expression but may rather be dependent on the mode of Nectin-4 engagement with T cells. Targeting Nectin-4 may offer an additional treatment approach for PDAC that merits clinical assessment. Expression of Nectin-4 is found in the embryo and placenta during fetal development and is rare in healthy adult tissues, but it is often overexpressed in tumor tissues (25). It is an attractive therapeutic target in PDAC due to its highly tumor cell–specific expression. In a multicancer study, PDAC had the third highest Nectin-4 expression by IHC, after urothelial and breast cancer (20). In addition, Nectin-4 overexpression has frequently been linked to reduced survival in several other cancers (26). The antibody-drug conjugate EV, which binds Nectin-4 and delivers a microtubule disrupting agent, proved beneficial as a second-line treatment after platinum-based chemotherapy and PD-1– or PD-L1–blockade in locally advanced or metastatic urothelial carcinoma (27). The EPIC trial (NCT05915351) is currently investigating the efficacy of EV in metastatic PDAC (28). PDOs recapitulate the genetic landscape of their parental tumors and have high predictive accuracy when studying patient-specific responses (29–32). Using several PDOs derived from patients with PDAC, we showed antitumor efficacy of EV, particularly in several chemoresistant PDOs, indicating the potential of EV as a therapeutic alternative for patients with PDAC. In conclusion, our study provides further evidence for the involvement of the TIGIT axis in PDAC immune evasion and uncovers Nectin-4 instead of CD155 as the most clinically relevant ligand, which also presented as a strong risk factor for reduced overall survival. While supporting TIGIT blockade as an immunotherapeutic strategy, this study provides the rationale to target Nectin-4 in PDAC, which merits clinical evaluation.

## Methods

### Sex as a biological variable.

Our study examined samples from male and female patients with PDAC, and our findings are expected to be relevant for more than one sex.

### Patient samples.

All human samples were obtained from patients of the University Hospital Carl Gustav Carus. For flow cytometry and in vitro studies, fresh tumor specimens and matched blood samples were collected from patients with PDAC, who underwent surgery between 2018 and 2024. Blood was drawn before surgical incision, and fresh tumor specimens were collected immediately after resection and evaluated by a trained pathologist. For IHC staining, formalin-fixed and paraffin-embedded PDAC tissue sections were obtained from the Institute of Pathology of the University Hospital Dresden. These samples matched fresh tumors, which had been processed for flow cytometry. The clinical stage of the tumors were classified according to the TNM system (UICC; Edition 8).

### Multicolor flow cytometry.

Single-cell suspensions of blood and PDAC samples for flow cytometry were prepared as described previously (7). Cells were stained both extra- and intracellularly with monoclonal antibodies listed in Supplemental Table 6. Cells were fixed and permeabilized for intracellular staining with eBioscience FOXP3/Transcription Factor Staining Buffer Set (Thermo Fisher) according to the manufacturer’s protocol. Flow cytometry was performed using a LSR Fortessa flow cytometer (BD Biosciences, RRID:SCR_018655). Data were analyzed using FlowJo v10.7.1 (Treestar, Ashland, OR, RRID:SCR_008520). A minimum number of 200 cells was set as a prerequisite for the subset analysis. Each patient was analyzed individually according to a previously shown gating hierarchy (7).

### IHC.

Sections of formalin-fixed, paraffin-embedded PDAC tissues were deparaffinized and rehydrated. Antigen retrieval was performed by boiling the slides in sodium citrate buffer (pH 6.0), and DAKO Protein Block (Agilent) was used to block nonspecific binding. Anti-CD155 (ab123252, Abcam, RRID:AB_10975440) or anti-Nectin-4 (PA5-30837, Invitrogen, RRID:AB_2548311), both 1:200 in Dako Antibody Diluent (Agilent), were applied at 4°C overnight. Anti-rabbit SignalStain boost IHC Detection Reagent (Cell Signaling Technology) was used as a secondary detection antibody for 30 minutes at room temperature. The ImmPACT DAB Peroxidase Substrate Kit (Vector Labs) was used according to the manufacturer’s instructions for a chromogenic reaction. Counterstaining was performed with Mayer’s hematoxylin (Clin-Tech). A trained pathologist calculated the IRS by multiplying the staining intensity (score 0–3) with the proportion of positive tumor cells (score 0–4) (33). An IRS of 0–4 was considered as low expression, and an IRS of 6–12 as high. Representative images were taken with the ECHO Revolve microscope (RRID:SCR_026523) at 10× magnification.

### Single-cell RNA-Seq.

The scRNA-Seq data has previously been published (17). Samples were collected from 26 patients with PDAC, including 17 primary tumors and 9 liver metastases at the Perlmutter Cancer Center at NYU Langone Health after obtaining informed written consent. Single-cell suspensions were processed for 10x Genomics by the Genome Technology Center at the NYU School of Medicine per the manufacturer’s guidelines. Sequencing results were demultiplexed and converted to FASTQ format using Illumina bcl2fastq software. The 10x Genomics Cell Ranger 5.0.1 software suite (34) was used to perform sample de-multiplexing, barcode processing, and single-cell 3′ gene counting aligned to the hg38/GRCh38 reference genome. Only confidently mapped, non-PCR duplicates with valid barcodes and unique molecular identifiers were used to generate the gene-barcode matrix. Clusters were identified based on common marker genes, for various cell types, the most prominent of which are listed here: CD8^+^ T cells (*CD3E*, *CD8*), Tconvs (*CD3E*, *CD4*, *FOXP3*-neg), Tregs (*CD3E*, *CD4*, *FOXP3*), NK (*NCAM1*), B/Plasma (*CD79A*), Mast (*KIT*), MDSC (*S100A8*, *S100A9*, *S100A12*), monocytes (*FGCR3A*, *CDKN1C*), macrophages (*CD68*), pDC (*LILRA4*, *PLD4*), cDC (*CD1C*), iCAFs (*C3*, *C7*, *CFD*, *PTGDS*), myCAFs (*ACTA2*, *MMP11*, *COL10A1*-neg), endothelial (*PECAM1*, *VWF*), and epithelial (*KRT19*). InferCNV version 1.8.1 was run at a sample level to differentiate between malignant and nonmalignant pancreatic epithelial cells. Further analyses, including the generation of the dot plots and violin plots, were performed using Seurat (35) and scooter (36). For more detailed information on the sample set, sample preparation, and initial data processing, see reference (17).

### TCGA data analysis.

The PAAD dataset (https://portal.gdc.cancer.gov/) was analyzed for the correlation between different genes of interest, assessing the Spearman’s rank correlation coefficients. Standardized expression levels from 146 patients with PDAC were depicted in a heatmap, ranked by *PVRL4* expression, using GraphPad Prism 9.3.1 (San Diego, California USA, www.graphpad.com, RRID:SCR_002798).

### In vitro T cell assay.

Cryopreserved PBMC samples from patients with PDAC who underwent neoadjuvant chemotherapy (FOLFIRINOX or Gemcitabine plus Paclitaxel) were thawed in prewarmed DPBS (Sigma Aldrich) supplemented with fetal calf serum (Gibco). Pan T cells were isolated trough negative selection using the Pan T cell Isolation Kit (Miltenyi). One day prior seeding, 96-well plates (U-bottom, Greiner Bio-One) were coated overnight with 10 μg/mL anti-CD3 (BioLegend, RRID:AB_11146991), 10 μg/mL anti-CD28 (BioLegend, RRID:AB_11148949) together with 20 μg/mL recombinant Nectin-4 protein (R&D Systems). Pan T cells were plated at 1 × 10^5^ cells per well in T cell medium — RPMI-1640 (Gibco) supplemented with 10% human AB serum (Sigma Aldrich), 2.5% HEPES (Gibco) and 1% Pen/Strep (Gibco) — and incubated for 72 hours. In addition, soluble recombinant Nectin-4 protein was added to the culture medium at a final concentration of 20 μg/mL at the time of plating. No medium change was performed during the incubation period. The supernatants were collected and cytokines IFN-γ and TNF-α were measured using the Th1, Th2, and Th17 CBA kit (BD) on a LSR Fortessa (BD Biosciences, RRID:SCR_018655).

### Gene expression analysis.

Total mRNA was obtained from each PDO line using RNeasy Kit (QUIAGEN) and genomic DNA was digested using RNase-free DNase Kit (QUIAGEN). qPCR was performed with GoTaq qPCR Kit (Promega) on a StepOnePlus RT PCR System (Applied Biosystems). *PVRL4* expression was analyzed using cDNA synthesized from each PDO line with the MultiScribe Reverse Transcriptase Kit (Applied Biosystems). The genes *GAPDH* and *RPL13* were chosen as internal controls. For both control genes and the target gene *PVRL4*, QuantiNova LNA PCR Assays (QUIAGEN) were used (HS_GAPDH_1799381, HS_RPL13_1769191, HS_NECTIN4_1411019). Amplification reactions were performed in duplicates and relative gene expression was evaluated using comparative ΔCT method.

### Western blot.

Proteins were obtained from each PDO line by resuspending cell pellets in lysis buffer (50 mM Tris-HCl with pH 8, 150 mM NaCl, 1% NP-40, 0.5% sodium deoxycholate, 0.1% SDS) with protease and phosphatase inhibitors (Thermo Fisher Scientific). Lysates were loaded in 20 μg protein per well and separated by electrophoresis on a SDS-PAGE gel (Invitrogen) and then transferred onto a PVDF membrane. The membrane was incubated overnight at 4°C with Nectin-4 antibody (17402, Cell Signaling Technology, RRID:AB_2798785). GAPDH antibody (2118S, Cell Signaling Technology, RRID:AB_561053) and horseradish peroxidase-conjugated secondary antibody (7074S, Cell Signaling Technology, RRID:AB_2099233) were incubated for 1 hour at room temperature.

### Human PDAC organoids.

PDOs were generated from surgical resection specimens as described previously (29). Briefly, tumor samples were cut into small pieces and digested using dispase II (2.5 mg/mL, Roche) and collagenase II (0.625 mg/mL, Sigma-Aldrich) in DMEM/F12+++ medium — DMEM/F12 (Invitrogen) supplemented with 1× HEPES (Invitrogen), 1× Pen/Strep (Invitrogen) and 1× GlutaMAX (Invitrogen) — at 37°C. The cell pellet was resuspended in GFR Matrigel (Corning). PDAC PDOs were cultivated in PDAC organoid medium DMEM/F12+++ supplemented with Wnt3a-conditioned medium (50% v/v), Noggin-conditioned medium (10% v/v), R-spondin-conditioned medium (10% v/v), B27 (1×, Invitrogen), nicotinamide (10 mM, Sigma-Aldrich), gastrin (1 nM, Sigma-Aldrich), N-acetyl-L-cysteine (1 mM, Sigma-Aldrich), Primocin (1 mg/mL, InvivoGen), recombinant murine epidermal growth factor (mEGF, 50 ng/mL, Invitrogen), recombinant human fibroblast growth factor 10 (hFGF-10, 100 ng/mL, PeproTech), A-83-01 (0.5 μM, Tocris Bioscience), and N2 (1×, Invitrogen). For the first 2–6 passages, PDAC PDOs were supplemented with Y-27632 (10 μM, Sigma-Aldrich). Depending on the growth rate, PDAC PDOs were passaged one to 2 times a week with a ratio of 1:2 to 1:4.

### In vitro drug assays.

PDAC PDOs were passaged on day 0 and supplemented with dispase II (1 mg/mL, Roche) on day 1, following a 2 hours incubation to enzymatically digest the matrigel. PDAC PDOs in suspension were then filtered by size (pluriStrainer, pluriSelect). Organoids between 20–50 μm in size were plated as triplicates in 384-well plates (μClear white, Greiner Bio-One) in 15 μL 75% Matrigel. Chemotherapeutics were provided by the local pharmacy department at the University Hospital Dresden and used as described earlier (29). Briefly, the dilution step *n* for FOLFIRINOX contained 10 μM irinotecan plus 35 μM oxaliplatin plus 35 μM 5-FU. The dilution step *n* for Gem/Pac contained 11.2 μM gemcitabine plus 7.2 μM paclitaxel. The antibody-drug conjugate EV was used in serial dilution from 0.1 mM to 600 mM. To evaluate the effect of single and combination drugs, cell viability was measured using CellTiter Glo 3D (Promega) after a total treatment of 6 days. Luminescence was measured using a Varioskan LUX (Thermo Fisher Scientific). Every single and combination drug treatment was performed 2 times in independent experiments and averaged for dose response curves and following analyses.

### Live cell imaging.

PDAC PDOs in suspension were obtained as described above. PDAC PDOs were filtered by size, and organoids between 50 and 100 μm were plated as duplicates in 384-well plates (Corning) in 15 μL 15% Matrigel. Based on the dose response curves for each single and combination drug, mean IC_50_ values were calculated and the next lower previously applied concentration was used for treatment (FOLFIRINOX n/9, Gem/Pac n/4, EV 10 μg/mL). After 2 days of incubation, Caspase-3 dye BioTracker NucView 488 Green (Thermo Fisher Scientific) was added to each well with a final concentration of 5 μM. PDOs were imaged on day 3 with Operetta CLS (Perkin Elmer, RRID:SCR_018810).

### Statistics.

Data are shown as the median in scatter plots or mean in bar graphs. Unpaired or paired 2-tailed *t* tests with Holm-Šídák correction were used as applicable. Wilcoxon signed-rank test was used for comparison of expression level within scRNA-Seq data. Kaplan-Meier plots for survival analysis of R0-resected patients (R0 indicates no microscopic tumor at the margins of resected specimen) were generated with GraphPad Prism 9.3.1 and evaluated by the log-rank test. A multivariate Cox proportional-hazards regression considering T, N, and M stage, resection margin (R), neoadjuvant chemotherapy, age, and sex was used to define HRs for intratumoral CD155 or Nectin-4 expression by using R Environment for Statistical Computing. Patients who died within 30 days after surgery were excluded from survival analysis. Fisher’s exact test was used to compare characteristics of the control and PDAC cohort and CD155 or Nectin-4 IRS distributions as a function of clinical characteristics. Pearson correlation coefficient was used to analyze the correlation between the expression of TIGIT, CD226, and CD96 within the different T cell subsets or within the scRNA-Seq expression data. *P* ≤ 0.05 was considered statistically significant, except for the correlation matrices, where *P* ≤ 0.01 was considered significant to account for multiple comparisons. All PDO lines were analyzed in 2 independent experiments for each single and combination drug. Values were averaged, and SD was calculated. Using GraphPad Prism, dose response curves were generated, and IC_50_ values and AUC were determined. For AUC *z* score normalization, relative AUC (AUCrel) was calculated from the quotient of AUC of dose response curve normalized to AUC 100%, which represents the relative viability as 100%. The formula z = (x – μ)/σ was used, where x is the mean AUCrel from the PDO line tested in 2 individual experiments, μ is the mean AUCrel from all PDO lines analyzed, and σ is the SD from all PDO lines analyzed. For comparison of cytokine expression of T cells, unpaired 2-tailed *t* test with Welch’s correction were applied. GraphPad Prism (GraphPad Software, RRID:SCR_002798) was used, and *P* ≤ 0.05 was considered statistically significant.

### Study approval.

All human samples were obtained from patients of the University Hospital Carl Gustav Carus, who gave written consent to a protocol approved by the Ethics Committee of the Technische Universität Dresden (no. EK446112017).

### Data availability.

All Supporting data values associated with the main manuscript and supplement material, including values for all data points shown in graphs and values behind any reported means, are provided in the Supporting Data Values file. The raw scRNA-Seq data used for this project are available under GEO accession no. GSE205013 (17). The raw flow cytometry data of this study are available from the corresponding author upon reasonable request.

## Author contributions

MH, LS, and AMS conceived this study. MH, CB, EAK, and JG developed the methodology. MH and CB performed the experiments and analyzed the data. EAK, SK, and DMS analyzed the scRNA-Seq data. FB and DES established patient-derived organoids. DD, LN, CR, and FH supported data acquisition. US evaluated and scored the IHC staining. MH, CB, SC, LS, and AMS curated the data. LS and AMS acquired funding. JW provided the research facilities. MH and CB wrote the manuscript. All authors reviewed the manuscript. Among both co–first authors, authorship order was determined according to contribution to study conception by MH.

## Funding support

- Jung Stiftung (LS)
- Monika Kutzner Stiftung (AMS)
- German Research Foundation (DFG; SE2980/5-1; LS)
- German Cancer Consortium (DKTK; AMS)
- Medical Faculty Carl Gustav Carus Technische Universität Dresden (MH, AMS)
- Else Kröner-Fresenius-Stiftung (Else Kröner Clinician Scientist Professorship; LS)
- Federal Ministry of Education and Research (Advanced Clinician Scientist Program CAMINO Dresden; AMS)

## Supplementary Material

Supplemental data

Unedited blot and gel images

Supporting data values

## Version 1 12/09/2025

In-Press Preview

## Version 2 01/23/2026

Electronic publication
