# Supplementary material for: Nectin-4 reduces T cell effector function and is a therapeutic target in pancreatic cancer
Source: JCI Insight. 2025 Dec 9;11(2):e194290. doi: 10.1172/jci.insight.194290 (PMC12892910; doi:10.1172/jci.insight.194290)
Supplement: Supplemental data [file jciinsight-11-194290-s128.pdf]

SUPPLEMENTARY MATERIAL

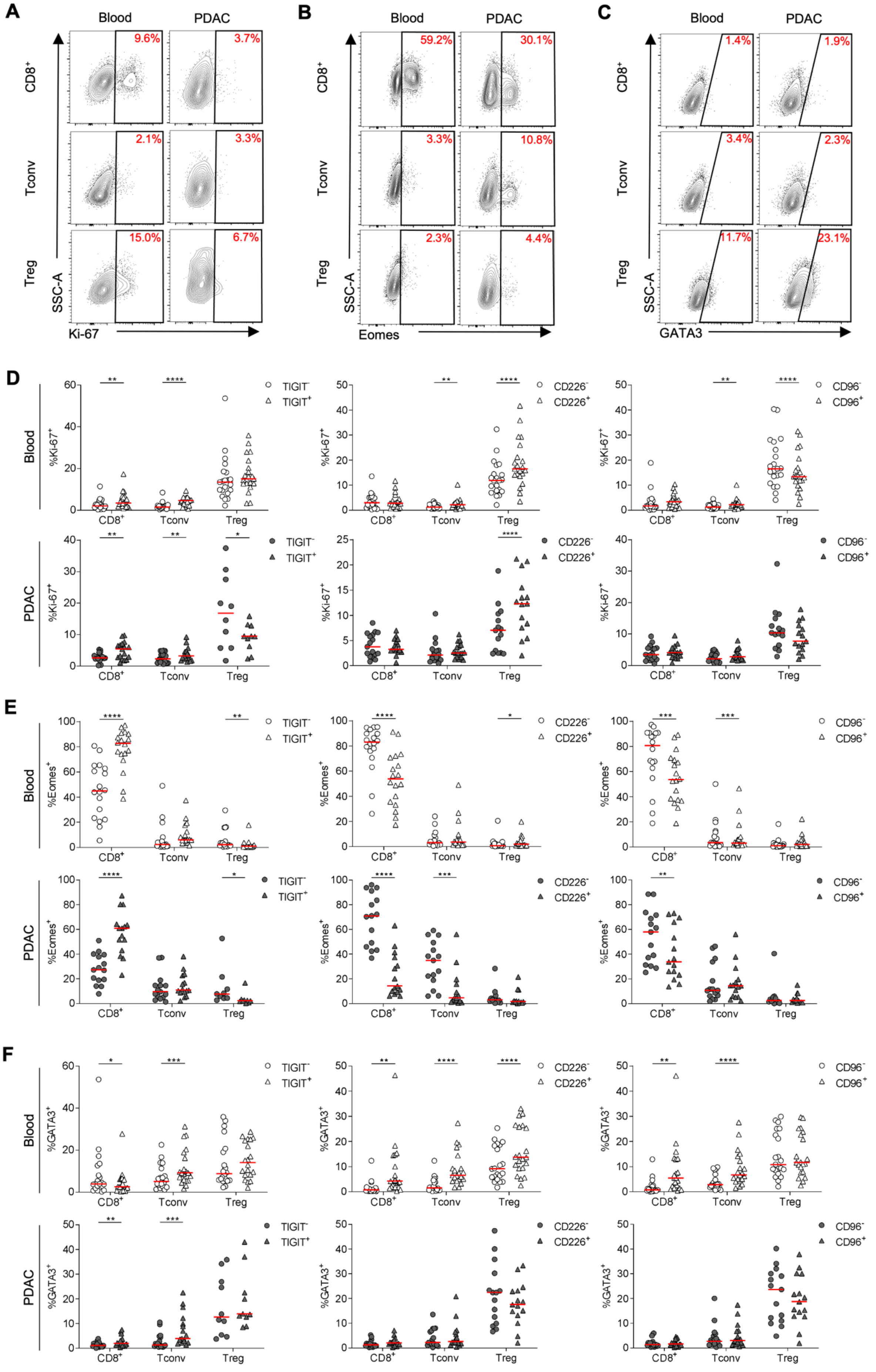

Supplementary Figure S1. (**A**) Representative contour flow plots for expression of Ki-67, (**B**) Eomes, and (**C**) GATA3 by indicated T cell subsets in blood and PDAC. The percentage of positive cells is indicated. (**D**) Percentage of Ki-67, (**E**) Eomes, and (**F**) GATA3 expression as a function of TIGIT, CD226 or CD96 expression (from left to right) for indicated T cell subsets in blood (top) and PDAC (bottom). Each point represents data from one patient. Medians are shown as horizontal red lines. Unpaired two-sided t tests with Holm-Šídák correction. *, $P < 0.05$; **, $P < 0.01$; ***, $P < 0.001$; ****, $P < 0.0001$.

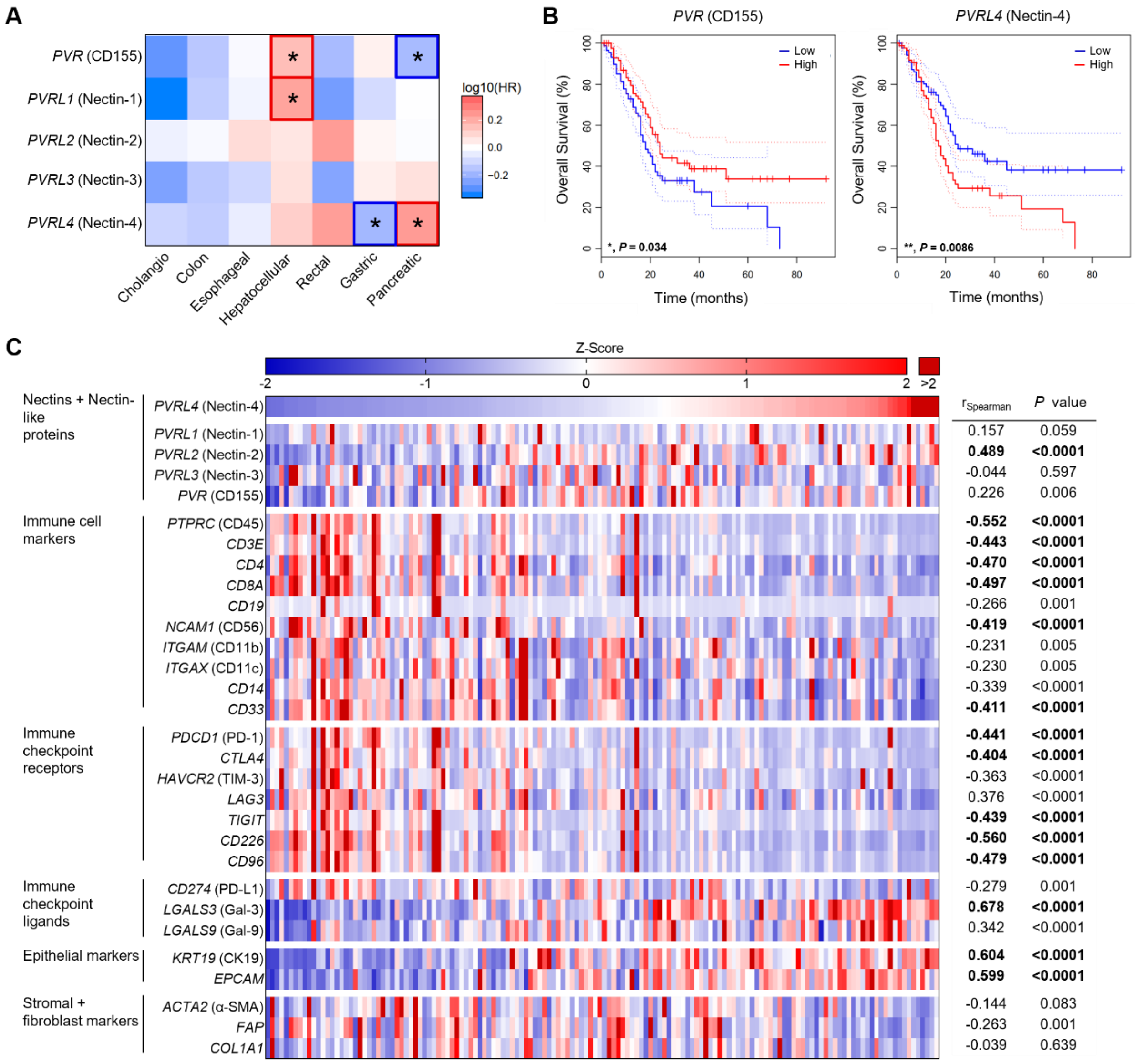

Supplementary Figure S2. (**A**) Heatmap generated with GEPIA2 showing overall survival hazard ratios (HR) for expression of *PVR, PVRL1, PVRL2, PVRL3* and *PVRL4* in indicated gastrointestinal carcinomas. *, *P* < 0.05. (**B**) Kaplan-Meier analysis generated with GEPIA2 for low versus high expression of *PVR* (left) or *PVRL4* (right) with the median as the threshold. HR and *P*-value of log rank test are indicated. (**C**) Heatmap showing standardized expression levels of genes of interest (Z-scores) in PDAC TCGA data. Each column represents one patient, ranked by *PVRL4* expression. The table shows the Spearman's rank correlation coefficients (rSpearman)

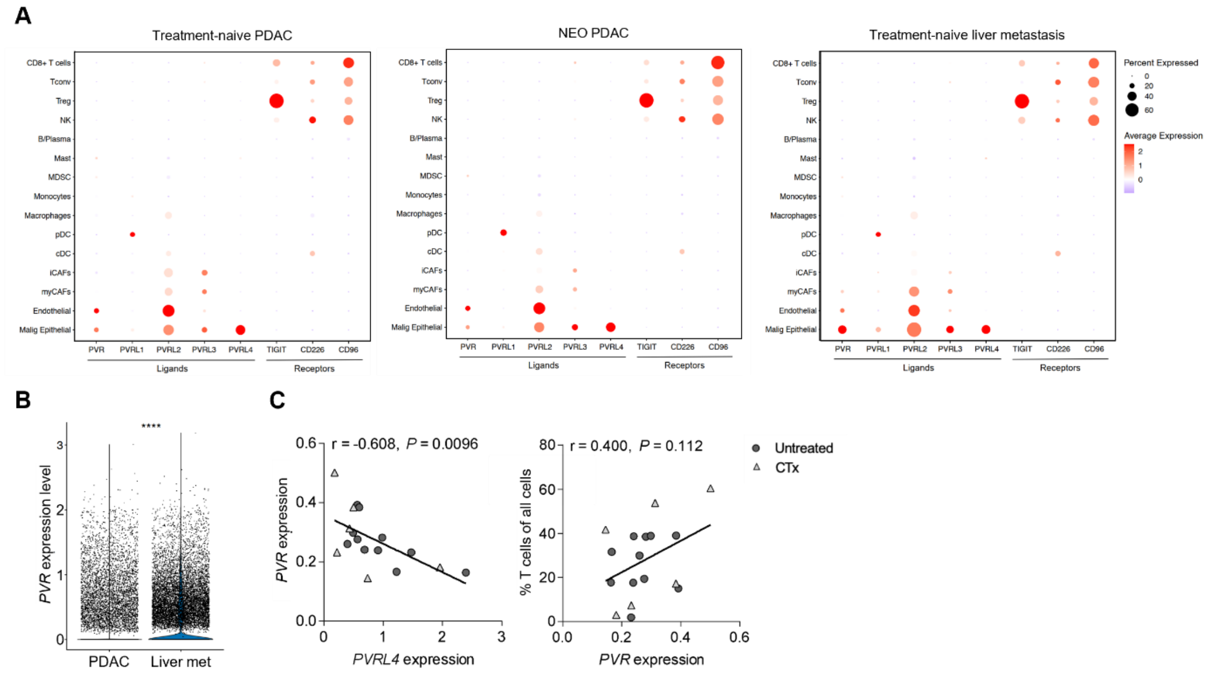

Supplementary Figure S3. (**A**) Dot plots depicting gene expression of TIGIT family receptors and ligands in several compartments within treatment-naive PDAC (n = 11, left), chemotherapeutically treated PDAC (n = 6, middle) and treatment-naive liver metastases (n = 9, right). The dot size represents the percent of cells expressing the gene and the color represents the average expression within those cells. (**B**) Violin plot of the expression of *PVR* expression in all malignant epithelial cells in primary PDAC (n = 11) compared to PDAC liver metastases (n = 9, right) from treatment-naive patients. Wilcoxon signed-rank test for comparison of expression level. ****, $P < 0.0001$. (**C**) Scatterplots showing the correlation between *PVR* and *PVRL4* expression by malignant epithelial cells and between *PVR* expression in malignant epithelial cells and T cell infiltration as a percentage of T cells among all analyzed cells per sample in treatment-naive (n = 11) and chemotherapeutically treated (n = 6) primary PDAC. Pearson correlation coefficients and P-values are depicted. Each dot represents one sample.

Supplementary Table S1. Patient characteristics of flow cytometry cohort.

**Variable**	**TIGIT (n = 84) n (%)**	**CD226 and CD96 (n = 19) n (%)**
**Age (Years)**		
Median (Range)	68 (35-84)	70 (35-80)
**Sex**		
Male	40 (48)	12 (63)
Female	44 (52)	7 (37)
**T Stage**		
1	6 (7)	2 (10)
2	42 (50)	6 (32)
3	32 (38)	11 (58)
4	4 (5)	0 (0)
**N Stage**		
0	23 (27)	6 (31.5)
1	34 (41)	7 (37)
2	27 (32)	6 (31.5)
**M Stage**		
0	70 (83)	17 (89)
1	14 (17)	2 (11)
**UICC**		
I	10 (12)	2 (11)
II	39 (46)	10 (52)
III	21 (25)	5 (26)
IV	14 (17)	2 (11)
**Margin**		
R0	65 (77)	16 (84)
R1	14 (17)	2 (11)
Rx	5 (6)	1 (5)
**NeoCTx**		
No	55 (65)	14 (74)
Yes	29 (35)	5 (26)

Supplementary Table S2. Pearson correlation coefficient (r) and *P*-values of correlation between TIGIT, CD226 and CD96 expression of indicated blood T cell subsets in the flow cytometry cohort.

		TIGIT of			CD226 of			CD96 of		
		**CD8$^+$**	**Tconv**	**Treg**	**CD8$^+$**	**Tconv**	**Treg**	**CD8$^+$**	**Tconv**	**Treg**
**TIGIT of**	**CD8$^+$**		r = 0.299<br>*P* = 0.147	r = 0.560<br>*P* = 0.004	r = -0.079<br>*P* = 0.706	r = 0.159<br>*P* = 0.449	r = -0.003<br>*P* = 0.990	r = 0.123<br>*P* = 0.557	r = 0.459<br>*P* = 0.021	r = 0.295<br>*P* = 0.152
	**Tconv**	r = 0.299<br>*P* = 0.147		r = 0.427<br>*P* = 0.033	r = 0.208<br>*P* = 0.318	r = 0.129<br>*P* = 0.540	r = 0.169<br>*P* = 0.418	r = 0.085<br>*P* = 0.686	r = 0.083<br>*P* = 0.694	r = 0.128<br>*P* = 0.543
	**Treg**	r = 0.560<br>*P* = 0.004	r = 0.427<br>*P* = 0.033		r = 0.442<br>*P* = 0.027	r = 0.470<br>*P* = 0.018	r = 0.366<br>*P* = 0.072	r = 0.096<br>*P* = 0.649	r = 0.277<br>*P* = 0.180	r = 0.150<br>*P* = 0.473
**CD226 of**	**CD8$^+$**	r = -0.079<br>*P* = 0.706	r = 0.208<br>*P* = 0.318	r = 0.442<br>*P* = 0.027		r = 0.747<br>*P* = 0.0002	r = 0.377<br>*P* = 0.063	r = -0.300<br>*P* = 0.145	r = -0.111<br>*P* = 0.599	r = -0.004<br>*P* = 0.984
	**Tconv**	r = 0.159<br>*P* = 0.449	r = 0.129<br>*P* = 0.540	r = 0.470<br>*P* = 0.018	r = 0.747<br>*P* = 0.0002		r = 0.551<br>*P* = 0.004	r = -0.155<br>*P* = 0.460	r = 0.044<br>*P* = 0.836	r = -0.224<br>*P* = 0.317
	**Treg**	r = -0.003<br>*P* = 0.990	r = 0.169<br>*P* = 0.418	r = 0.366<br>*P* = 0.072	r = 0.377<br>*P* = 0.063	r = 0.551<br>*P* = 0.004		r = 0.130<br>*P* = 0.537	r = 0.166<br>*P* = 0.427	r = 0.191<br>*P* = 0.360
**CD96 of**	**CD8$^+$**	r = 0.123<br>*P* = 0.557	r = 0.085<br>*P* = 0.686	r = 0.096<br>*P* = 0.649	r = -0.300<br>*P* = 0.145	r = -0.155<br>*P* = 0.460	r = 0.130<br>*P* = 0.537		r = 0.520<br>*P* = 0.008	r = 0.440<br>*P* = 0.028
	**Tconv**	r = 0.459<br>*P* = 0.021	r = 0.083<br>*P* = 0.694	r = 0.277<br>*P* = 0.180	r = -0.111<br>*P* = 0.599	r = 0.044<br>*P* = 0.836	r = 0.166<br>*P* = 0.427	r = 0.520<br>*P* = 0.008		r = 0.875<br>*P* < 0.0001
	**Treg**	r = 0.295<br>*P* = 0.152	r = 0.128<br>*P* = 0.543	r = 0.150<br>*P* = 0.473	r = -0.004<br>*P* = 0.984	r = 0.034<br>*P* = 0.872	r = 0.191<br>*P* = 0.360	r = 0.440<br>*P* = 0.028	r = 0.875<br>*P* < 0.0001	

Supplementary Table S3. Pearson correlation coefficient (r) and *P*-values of correlation between TIGIT, CD226 and CD96 expression of indicated PDAC-infiltrating T cell subsets in flow cytometry.

		**TIGIT of**			**CD226 of**			**CD96 of**		
		**CD8$^+$**	**Tconv**	**Treg**	**CD8$^+$**	**Tconv**	**Treg**	**CD8$^+$**	**Tconv**	**Treg**
**TIGIT of**	**CD8$^+$**		r = 0.682<br>*P* = 0.001	r = 0.621<br>*P* = 0.005	r = -0.625<br>*P* = 0.003	r = -0.379<br>*P* = 0.099	r = -0.034<br>*P* = 0.891	r = 0.190<br>*P* = 0.422	r = 0.076<br>*P* = 0.750	r = 0.041<br>*P* = 0.869
	**Tconv**	r = 0.682<br>*P* = 0.001		r = 0.782<br>*P* < 0.0001	r = -0.406<br>*P* = 0.076	r = -0.478<br>*P* = 0.033	r = -0.345<br>*P* = 0.148	r = 0.469<br>*P* = 0.037	r = 0.326<br>*P* = 0.160	r = 0.236<br>*P* = 0.331
	**Treg**	r = 0.621<br>*P* = 0.005	r = 0.782<br>*P* < 0.0001		r = -0.290<br>*P* = 0.229	r = -0.328<br>*P* = 0.170	r = -0.486<br>*P* = 0.035	r = 0.226<br>*P* = 0.353	r = 0.291<br>*P* = 0.228	r = 0.209<br>*P* = 0.390
**CD226 of**	**CD8$^+$**	r = -0.625<br>*P* = 0.003	r = -0.406<br>*P* = 0.076	r = -0.290<br>*P* = 0.229		r = 0.565<br>*P* = 0.009	r = 0.142<br>*P* = 0.563	r = 0.026<br>*P* = 0.914	r = 0.279<br>*P* = 0.233	r = 0.318<br>*P* = 0.185
	**Tconv**	r = -0.379<br>*P* = 0.099	r = -0.478<br>*P* = 0.033	r = -0.328<br>*P* = 0.170	r = 0.565<br>*P* = 0.009		r = 0.617<br>*P* = 0.005	r = -0.209<br>*P* = 0.376	r = 0.025<br>*P* = 0.917	r = -0.111<br>*P* = 0.651
	**Treg**	r = -0.034<br>*P* = 0.891	r = -0.345<br>*P* = 0.148	r = -0.486<br>*P* = 0.035	r = 0.142<br>*P* = 0.563	r = 0.617<br>*P* = 0.005		r = -0.284<br>*P* = 0.239	r = -0.324<br>*P* = 0.176	r = -0.360<br>*P* = 0.130
**CD96 of**	**CD8$^+$**	r = 0.190<br>*P* = 0.422	r = 0.469<br>*P* = 0.037	r = 0.226<br>*P* = 0.353	r = 0.026<br>*P* = 0.914	r = -0.209<br>*P* = 0.376	r = -0.284<br>*P* = 0.239		r = 0.874<br>*P* < 0.0001	r = 0.751<br>*P* = 0.0002
	**Tconv**	r = 0.076<br>*P* = 0.750	r = 0.326<br>*P* = 0.160	r = 0.291<br>*P* = 0.228	r = 0.279<br>*P* = 0.233	r = 0.025<br>*P* = 0.917	r = -0.324<br>*P* = 0.176	r = 0.874<br>*P* < 0.0001		r = 0.835<br>*P* < 0.0001
	**Treg**	r = 0.041<br>*P* = 0.869	r = 0.236<br>*P* = 0.331	r = 0.209<br>*P* = 0.390	r = 0.318<br>*P* = 0.185	r = -0.111<br>*P* = 0.651	r = -0.360<br>*P* = 0.130	r = 0.751<br>*P* = 0.0002	r = 0.835<br>*P* < 0.0001	

Supplementary Table S4. Correlation of TIGIT ligands with markers of interest based on the TCGA data set. Spearman's rank correlation coefficient (r). r ≤ -0.4 or r ≥ 0.4 are highlighted in bold.

		PVRL1		***PVRL2***		***PVRL3***		***PVR***	
**Background**	**Gene**	**r**	***P*-Value**	**r**	***P*-Value**	**r**	***P*-Value**	**r**	***P*-Value**
Nectins and Nectin-like proteins	*PVRL1*			0.202	0.014	0.176	0.034	0.367	<0.0001
	PVRL2	0.202	0.014			-0.022	0.790	**0.486**	**<0.0001**
	PVRL3	0.176	0.034	-0.022	0.790			0.248	0.003
	PVRL4	0.157	0.059	**0.489**	**<0.0001**	-0.044	0.597	0.226	0.006
	PVR	0.367	<0,0001	**0.486**	**<0.0001**	0.248	0.003		
Immune cell markers	*PTPRC*	-0.131	0.114	-0.332	<0.0001	0.139	0.095	-0.094	0.258
	CD3E	-0.137	0.099	-0.198	0.017	0.027	0.742	-0.131	0.115
	CD4	-0.074	0.377	0.092	0.272	0.092	0.268	-0.107	0.200
	CD8A	-0.147	0.078	-0.278	0.001	0.084	0.314	-0.161	0.052
	CD19	-0.068	0.414	-0.102	0.220	0.018	0.833	-0.066	0.430
	NCAM1	-0.129	0.122	-0.217	0.009	0.153	0.065	-0.115	0.166
	ITGAM	-0.087	0.298	-0.142	0.088	0.074	0.377	-0.084	0.311
	ITGAX	-0.127	0.128	-0.009	0.915	-0.124	0.137	-0.149	0.074
	CD14	0.016	0.844	-0.083	0.318	-0.029	0.727	-0.100	0.229
	FCGR3A	0.063	0.451	-0.092	0.272	0.111	0.183	0.054	0.519
Immune checkpoint receptors	*PDCD1*	-0.103	0.216	-0.107	0.198	-0.054	0.519	-0.067	0.420
	CTLA4	-0.113	0.173	-0.151	0.070	-0.076	0.360	-0.055	0.510
	HAVCR2	0.017	0.843	-0.084	0.313	0.007	0.936	-0.041	0.623
	LAG3	-0.083	0.318	-0.142	0.087	0.018	0.827	-0.064	0.444
	TIGIT	-0.076	0.364	-0.222	0.007	-0.007	0.931	-0.092	0.270
	CD226	-0.123	0.141	-0.293	0.000	0.184	0.026	-0.103	0.217
	CD96	-0.160	0.053	-0.224	0.007	0.072	0.387	-0.125	0.133
Immune checkpoint ligands	*CD274*	0.186	0.025	-0.109	0.192	0.246	0.003	0.153	0.066
	LGALS3	0.128	0.124	**0.406**	**<0.0001**	0.092	0.267	0.274	0.001
	LGALS9	-0.025	0.761	**0.482**	**<0.0001**	-0.028	0.733	0.165	0.046
Epithelial marker	*KRT19*	0.301	<0.0001	**0.552**	**<0.0001**	0.099	0.233	0.328	<0.0001
	EPCAM	0.229	0.005	**0.435**	**<0.0001**	0.152	0.067	**0.409**	**<0.0001**
Stromal + Fibroblast marker	*ACTA2*	-0.114	0.172	-0.035	0.674	0.107	0.200	0.015	0.860
	FAP	0.063	0.450	-0.167	0.044	0.227	0.006	0.090	0.282
	COL1A1	0.051	0.537	0.004	0.966	0.087	0.299	0.016	0.846

Supplementary Table S5. Patient characteristics of CD155 and Nectin-4 immunohistochemistry cohort and association with IRS. Fisher's exact test was used to compare the IRS distribution for the respective clinicopathologic characteristic.

	CD155				Nectin-4			
**Variable**	**n**	**Low**	**High**	***P*-Value**	**n**	**Low**	**High**	***P*-Value**
**Age**								
<68 yrs	36	27	9	0.788	35	18	17	0.478
≥68 yrs	33	23	10		33	14	19	
**Sex**								
Male	33	25	8	0.600	32	13	19	0.342
Female	36	25	11		36	19	17	
**T Stage**								
1	6	3	3	0.547	6	1	5	0.371
2	32	23	9		32	15	17	
3	27	21	6		26	13	13	
4	4	3	1		4	3	1	
**N Stage**								
0	20	12	8	0.183	20	9	11	0.681
1	29	24	5		28	15	13	
2	20	14	6		20	8	12	
**M Stage**								
0	57	43	14	0.289	56	29	27	0.118
1	12	7	5		12	3	9	
**UICC**								
I	10	5	5	0.116	10	5	5	0.332
II	31	26	5		30	17	13	
III	16	12	4		16	7	9	
IV	12	7	5		12	3	9	
**NeoCTx**								
No	43	33	10	0.406	42	19	23	0.804
Yes	26	17	9		26	13	13	

Supplementary Table S6. Antibodies used for flow cytometry.

**Marker**	**Clone**	**Manufacturer**	**Catalog #**
CD45	HI30	BD Biosciences	563792
CD3	UCHT1	BD Biosciences	612940
CD8	SK1	BD Biosciences	564912
CD4	RPA-T4	BD Biosciences	560158
FOXP3	206D	BioLegend	320126
TIGIT	A15153G	BioLegend	372710
CD226	11A8	BioLegend	338312
CD96	6F9	BD Biosciences	562379
Ki-67	B56	BD Biosciences	563756
GATA3	L50-823	BD Biosciences	565449
Eomes	WD1928	Invitrogen	11-4877-42
