# Supplementary material for: Nectin-4 reduces T cell effector function and is a therapeutic target in pancreatic cancer
Source: JCI Insight. 2025 Dec 9;11(2):e194290. doi: 10.1172/jci.insight.194290 (PMC12892910; doi:10.1172/jci.insight.194290)
Supplement: Unedited blot and gel images [file jciinsight-11-194290-s129.pdf]

Western Blot membrane unedited

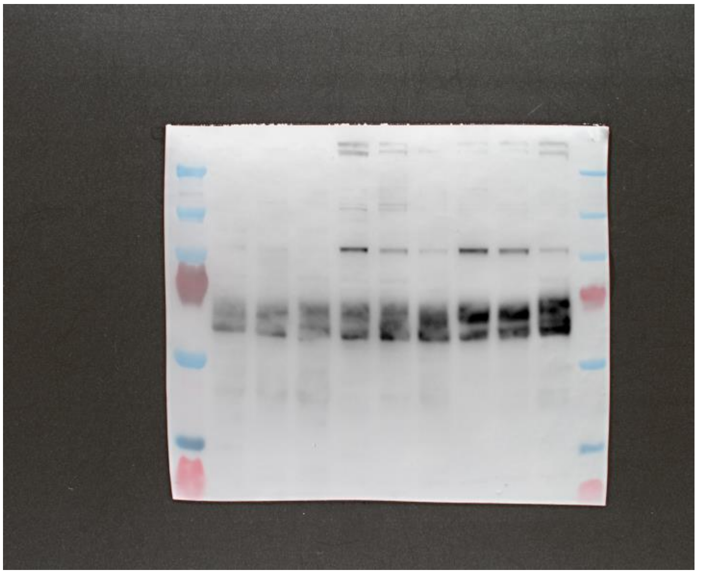

Full unedited blot for Figure 6C, antibody: anti-Nectin-4
#17402
Cell Signaling Technology
RRID:AB_2798785

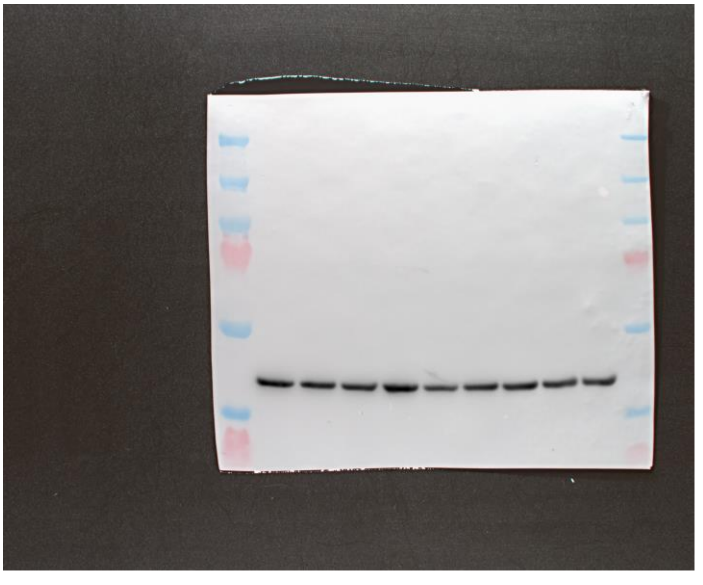

Full unedited blot for Figure 6C, antibody: anti-GAPDH
#2118S
Cell Signaling Technology
RRID:AB_561053

Horseradish peroxidase-conjugated secondary antibody
#7074S, Cell Signaling Technology, RRID:AB_2099233

Western Blot membrane marked

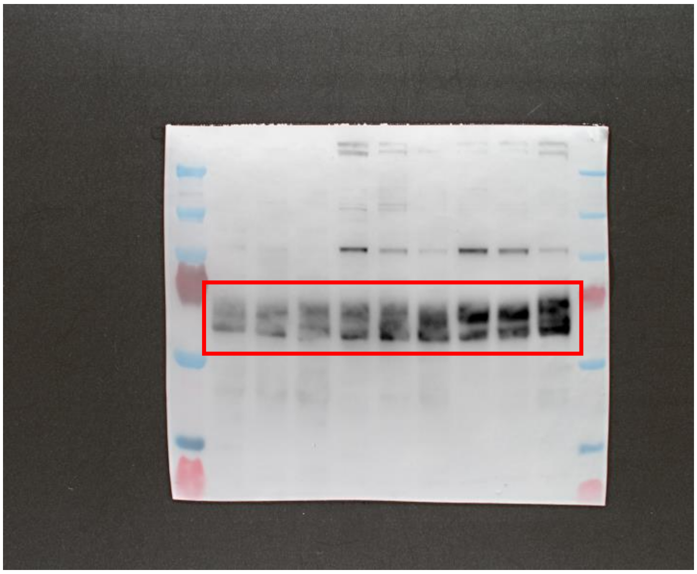

Figure 6C, antibody: anti-Nectin-4

The section used in the manuscript is marked in red (63 kDa).

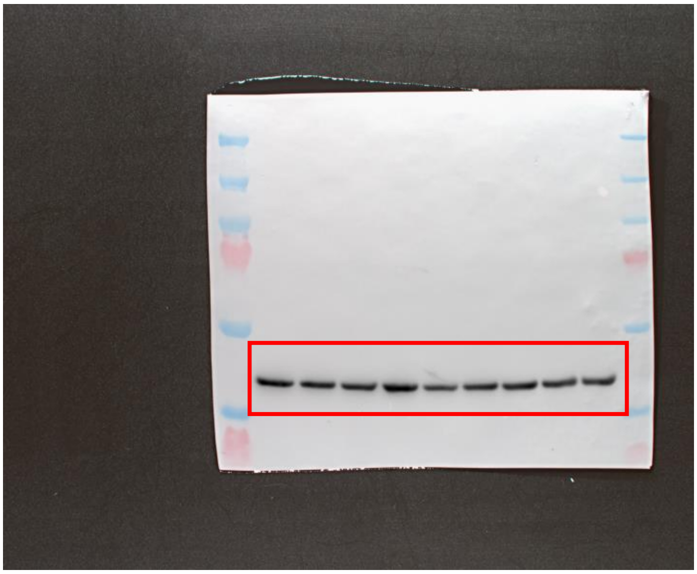

Figure 6C, antibody: anti-GAPDH

The section used in the manuscript is marked in red (37 kDa).
